# Un cas rare de carcinome épidermoïde de la langue, variante à cellules claires: à propos d’un cas

**DOI:** 10.11604/pamj.2022.43.200.37846

**Published:** 2022-12-22

**Authors:** Tiabi El Mehdi, Nassira Karich, Achraf Miry, Amal Bennani

**Affiliations:** 1 Service d’Anatomie Pathologique, Centre Hospitalier Universitaire Mohammed VI, Faculté de Médecine et de Pharmacie d’Oujda, Université Mohammed Premier, Oujda, Maroc

**Keywords:** Carcinome épidermoïde, cellules claires, immunohistochimie, cas clinique, Squamous cell carcinoma, clear cells, immunohistochemistry, case report

## Abstract

La variante à cellules claires du carcinome épidermoïde est une variante mal connue, très rare, et peu décrite dans la littérature, avec seulement 10 cas. Elle survient généralement au niveau de la cavité buccale, avec une prédominance féminine. Nous rapportons le cas d´un homme de 47 ans, qui s´est présenté avec une masse bourgeonnante de la base de la langue, d´évolution rapide. Le diagnostic de carcinome épidermoïde, variante à cellules claires a été retenu sur des bases histologiques et immunohistochimiques.

## Abstract (en)

Clear cell squamous cell carcinoma is a poorly known, very rare, and poorly described variant of squamous cell carcinoma, with only 10 cases reported in the literature. It usually occurs in the oral cavity, with predominance of the female sex. We report the case of a 47-year-old man presenting with a rapidly growing budding mass in the base of the tongue. The diagnosis of clear cell squamous cell carcinoma was retained based on histological and immunohistochemical results.

## Introduction

Le carcinome épidermoïde (CE) est une tumeur maligne fréquente des kératinocytes épidermiques qui présente des degrés variables de différenciation. On le retrouve le plus souvent dans les zones exposées au soleil, notamment le cuir chevelu, et le visage. Cependant, c´est un processus qui se développe à la suite de plusieurs étapes. Dans sa variante à cellules claires, le carcinome épidermoïde demeure extrêmement rare, avec seulement 10 cas décrits dans la littérature anglaise [1]. Nous rapportons un cas de CE à cellules claires de la langue chez un homme de 47 ans.

## Patient et observation

**Informations relatives aux patients:** il s´agit d´un patient de 47 ans, qui présente des antécédents de tabagisme, et qui a consulté pour une tuméfaction de la langue évoluant depuis 4 mois, qui a débuté par une simple ulcération, et dont l´évolution était rapide, marquée par l´apparition d´une lésion bourgeonnante.

**Résultats cliniques:** l´examen clinique montre la présence d´une masse ulcéro-bourgeonnante de 6 cm de grand axe, située à la base de la langue.

**Figure 1:**
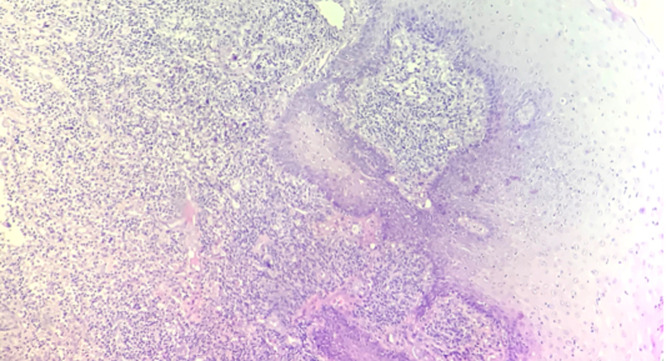
microphotographie montrant un processus carcinomateux infiltrant le derme (hématoxyline et éosine 100X)

**Figure 2:**
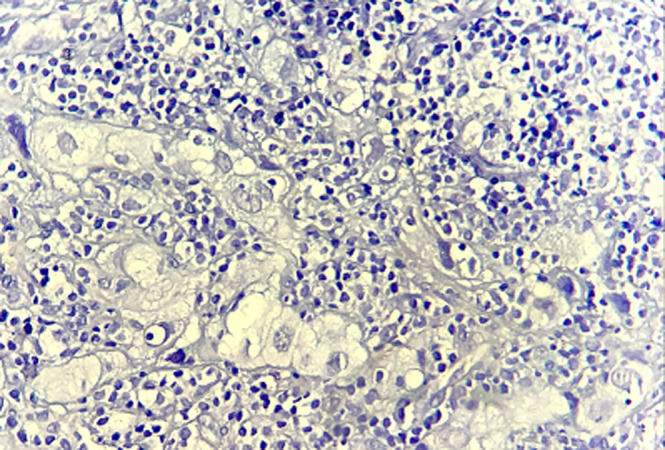
microphotographie avec plus fort grossissement montrant que la prolifération est constituée de grandes cellules, dotées d´un noyau volumineux, centré par un nucléole, et présentant des atypies franches, associées à un cytoplasme abondant vacuolaire (hématoxyline et éosine; 400X)

**Figure 3:**
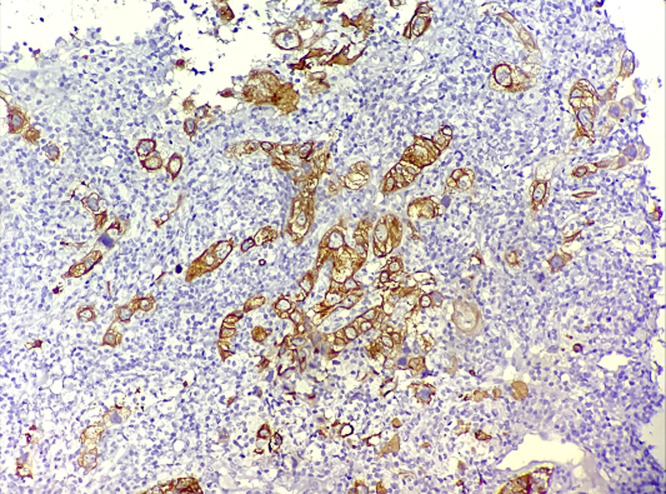
microphotographie montrant une expression cytoplasmique de la cytokératine 5/6 par les cellules tumorales

**Figure 4:**
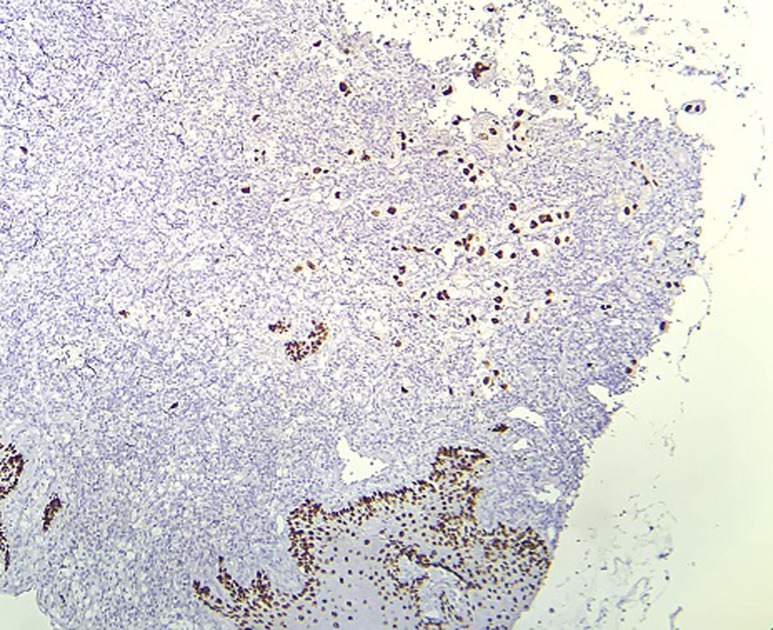
microphotographie montrant une expression nucléaire de la p63 par les cellules tumorales

**Démarche diagnostique:** le patient a par la suite bénéficié d´une biopsie de la masse. Sur le plan macroscopique, on a reçu un fragment de 0,6cm de grand axe. Histologiquement, il s´agit d´une muqueuse malpighienne siège d´un processus malin indifférencié, constitué de grosses cellules dotées d´un noyau volumineux, centré par un nucléole, et présentant des atypies franches associées à un cytoplasme abondant vacuolaire. Ces cellules sont disposées en des nappes diffuses et en quelques travées épaisses, sans formation de kératine (Figure 1 et Figure 2). Une coloration d´acide périodique de Schiff (PAS) a été réalisée, revenue négative, ne montrant pas de dépôt de mucine au sein des cellules tumorales. L´immunohistochimie montre un marquage positif des cellules tumorales par la CK5/6 et la P63. Absence de marquage par l´EMA, la CK7, le Melan A et l´HMB45 (Figure 3 et Figure 4).

**Intervention thérapeutique:** le patient a subi une excision chirurgicale avec des marges adéquates, suivie d´une radiothérapie.

**Suivi et résultats des interventions thérapeutiques:** l´évolution à court terme était favorable.

**Perspectives du patient:** le patient était satisfait par rapport à la prise en charge reçu.

**Consentement éclairé:** le patient a donné son consentement éclairé à propos de la publication de ce travail.

## Discussion

Le carcinome épidermoïde dans sa variante à cellules claires, est une entité très rare, décrite pour la première fois par Kuo en 1980 comme une tumeur cutanée. La plupart des cas ont été rapportés dans la région de la tête et du cou. Sa présence au niveau de la cavité buccale est exceptionnelle, et il s´agit généralement d´un néoplasme des glandes salivaires ou d´une tumeur métastatique [2]. L´aspect clair des cellules est généralement secondaire soit à la présence de glycogène, ou de mucine en intracytoplasmique, ou bien plus rarement à des défauts artéfactuels. La plupart des cas ont été observés chez des femmes âgées (6 CAS/10) [1], alors que notre cas a porté sur un homme d´âge moyen.

La présentation clinique semble être assez agressive car la lésion commence par un ulcère, qui évolue rapidement vers une grande masse exophytique, faisant plus de 10 cm, au bout de quelques mois d´évolution. L´étiologie demeure à nos jours méconnus. Néanmoins, certains facteurs ont été incriminés tels que le rayonnement ultra-violets (UV), l´immunosuppression chronique, la kératose actinique (lésion précurseur), l´albinisme, les cicatrices de brûlures, les ulcères chroniques et l´arsenic [3]. Les mutations impliquant des gènes (tels que TP53, CDKN2A, NOTCH1 et NOTCH2, EGFR et TERT) et des voies moléculaires (RAS/RAF/MEK/ERK et PI3K/AKT/mTOR) jouent un rôle important dans la pathogenèse [3]. Histologiquement, la prolifération se présente avec une architecture diffuse, plus rarement des travées ou des cordons. Les cellules tumorales sont volumineuses, dotées de noyau augmenté de taille, à chromatine fine, nucléolé par endroit, avec un cytoplasme abondant clair [3]. La présence de cellules claires nécessite la réalisation de colorations spéciales, notamment l´acide periodic de schiff (PAS), afin de déterminer la nature (glycogène, mucine, artéfact) [3]. Une étude immunohistochimique peut également s´avérer nécessaire au diagnostic, puisqu´elle va permettre d´éliminer les diagnostics différentiels (mélanome comme exemple), et de confirmer la nature malpighienne des cellules tumorales par la P63 et la CK5/6. Les principaux diagnostics différentiels sont décrits sur les tableaux ci-dessous (Tableau 1 et Tableau 2) [3,4].

**Tableau 1:** principaux diagnostics différentiels du carcinome épidermoïde à cellules claires [3,4]

Diagnostic		Épidémiologie	Histologie	Immunohistochimie-Coloration spéciale	Pronostic
Carcinome muco-épidermoïde (origine glande salivaire)		-Tumeur maligne des glandes salivaires la plus fréquente (adultes et enfants) -Rarement dans le tractus naso-sinusien, le nasopharynx, les poumons et la mandibule intra-osseuse	-Architecture: Solide-kystique-mixte. -Cellules: malpighiennes, mucocytes (aspect clair+)	IHC: CK5/6+; P63+; CK7+ PAS +	Dépend du grade: généralement de bon pronostic+
Carcinome à cellules claires (Glande salivaire)		-Rare, < 1 % -Âge moyen: 60ans. -Fréquent dans les glandes salivaires mineures (~80 %), en particulier la base de la langue, le palais, le plancher buccal	-Nids et cordons de cellules claires avec des limites cytoplasmiques distinctes. -Stroma hyalinisé.	CK7+, P63+ PAS+	Bon pronostic Récidive dans environs 11%
Carcinome odontogène à cellules claires		Rare, < 100 cas décrits Âge moyen: 60 ans. Site: Mandibule	Nids, cordons: cellules épithéliales claires, et basaloides. Stroma fibreux dense.	CK+, EMA+/-, PS100+	Taux de mortalité: 25%
Mélanome		Rare Age moyen: 61ans Fréquence des métastases	Cellules épithélioïdes, fusiformes et polymorphes. Nucléole proéminant. Mitoses +	PS100+, Melan A+, HMB45+, CK-.	Pronostic mauvais (métastases+)
Carcinome rénale à cellules claires métastatiques	Age moyen: 65ans		Nids compacts de cellules avec un cytoplasme clair et une membrane bien définie	PAX8+, PAX2+, CD10+ CK7-, CK20-	Pronostic médiocre

CK: cytokératine; EMA: antigène de la membrane épithéliale; PAS: acide périodique de Schiff; AML: actine musculaire lisse

**Tableau 2:** cas de carcinome épidermoïde à cellules claires de localisation buccale dans la littérature

Référence	Age/Sexe	Site	Étiologie	Immunohistochimie	Coloration spéciale	Pronostic
Kumar et al	70ans Femme	Région maxillaire antérieure	TABAC	Positif : EMA, CK8, CK18 Négatif :Vimentine S-100, HMB 45	Négatif :PAS Mucicarmin	Décès dans les 2 mois
Frazier et al.	59ans Femme	Région gingivale (cavité buccale)	__	__	PAS + Négative : PAS diastase, Mucicarmine	Perdue de vue
Nainani et a	52ans Homme	Cavité buccale	Alcoolique Non tabagique	Positive: EMA, CK8, CK18 Négative :Vimentine S-100, HMB 45	Négative: PAS, Mucicarmine, Oil red O	Décès dans les 3 mois
Romañach et al.	60ans Femme	Cavité buccale (palais)	__	Positive: CK, P63 Négative: Vimentine ,CD10	PAS+	Absence de récidive au bout de 12 mois
Kaliamoorthy et al	35ans Femme	Bord latéral de la langue	Alcoolique Non tabagique	Positive : CK Négative : Vimentine, HMB45, EMA	Négative : PAS, Mucicarmine	Perdue de vue
Devi et al.1	55ans Homme	Région postérieure du maxillaire	__	Positive : CK, EMA Negative: S-100, Vimentine	Négative: PAS, Mucicarmine,	Perdu de vue
Khoury et al.	66ans Femme	Masse de la langue	__	Positive: P63, CK5/6, CK Négative: PS100, AML	PAS+	Métastase pulmonaire (3mois après)
Kakoti et al.	59ans Homme	Joue droite	__	Positive: CK Négative: S100, EMA	PAS-	Perdu de vue
Ramani et al.	42ans Femme	Partie molle	__	Positive: CK Négative: CD117, EMA, S100, AML	Négative: PAS diastase Mucicarmine	Perdue de vue
Rutuja Narsing Mukkanwar et al	60ans Homme	Région postéro-latérale de la langue	TABAC	Positive : CK	Positive : PAS diastase Negative : Mucicarmine	Perdu de vue
Notre cas	47ans Homme	Base de la langue	TABAC	Positive : CK5/6, P63, CK Négative: S100, HMB45, CK7, EMA	PAS-	Bonne évolution à 1 mois, puis perdu de vue

Les modalités de traitement restent encore très peu connues, du fait de la rareté de cette entité d´une part, et de l´absence de suivi des patients comme ça été le cas pour 7 malades sur les 11 décrits dans la littérature. Certains auteurs préconisent un traitement chirurgical par ablation de la masse, suivi d´une radiothérapie. Cependant, aucune étude n´a démontré de réel consensus à nos jours. D´après les données recueillies pour les 11 patients, la variante à cellules claires du carcinome épidermoïde serait plus agressive et aurait un pronostic plus sombre que la variante classique [1-5]. Des études moléculaires sont nécessaires pour comprendre les paramètres biologiques, cliniques histopathologiques de ces cas.

## Conclusion

Le carcinome épidermoïde dans sa variante à cellules claires est une entité excessivement rare, avec très peu de cas décrits dans la littérature. L´évolution sur le plan clinique est rapide, avec apparition en quelques mois d´une masse exophytique. Le diagnostic est histologique et immunohistochimique. Le pronostic reste médiocre. Toutefois, d´autres études sont souhaitables afin de mieux détailler les moyens thérapeutiques adéquats.
